# What happened to the most frequent surgeries performed in the Brazilian Unified Health System during and after the COVID-19 pandemic? An analysis of 2 million procedures

**DOI:** 10.31744/einstein_journal/2025AO1399

**Published:** 2025-08-15

**Authors:** Altair da Silva Costa, Otavio Rocha Fiuza, Isabella Marques Vieira, Henrique Pavezi Fornazari, João Gabriel Bicudo Ting, Camila Kalim, Sabrina Menes Ares

**Affiliations:** 1 Hospital Israelita Albert Einstein São Paulo SP Brazil Hospital Israelita Albert Einstein, São Paulo, SP, Brazil.; 2 Universidade Federal de São Paulo São Paulo SP Brazil Universidade Federal de São Paulo, São Paulo, SP, Brazil.; 3 Hospital Israelita Albert Einstein Faculdade Israelita de Ciências da Saúde Albert Einstein São Paulo SP Brazil Faculdade Israelita de Ciências da Saúde Albert Einstein, Hospital Israelita Albert Einstein, São Paulo, SP, Brazil.; 4 Centro Universitário FMABC Santo André SP Brazil Centro Universitário FMABC, Santo André, SP, Brazil.

**Keywords:** Brazilian Unified Health System, Public Health, COVID-19, Health information management, Health services needs and demand, Pandemics, Surgical procedures, operative, Brazil

## Abstract

**Objective:**

To analyze changes in surgical volume across different specialties in Brazil before, during, and after the COVID-19 pandemic.

**Methods:**

This descriptive observational study compared surgical volumes for the specialties with the highest number of procedures each year from 2019 to 2023. Data were sourced from the Department of Informatics of the Unified Health System (DATASUS - TabNet).

**Results:**

A total of 2,117,383 surgical procedures across 13 operation types were analyzed. Between 2019 and 2020, surgical volume declined by 35%, with reductions ranging from -1% to -54% depending on the procedure. Although volumes increased by 2023, this was insufficient to address the backlog of delayed surgeries. Otorhinolaryngology and urology were the most affected specialties, while cholecystectomy saw a net post-pandemic increase.

**Conclusion:**

The COVID-19 pandemic significantly disrupted all analyzed surgical procedures in Brazilian Public Health System, resulting in a 35% overall decline. Despite partial recovery, the persistent surgical *deficit* remains a major challenge, reflecting patterns observed internationally.

## In Brief

COVID-19 significantly affected surgeries in Brazilian Unified Health System, leading to a 35% reduction between 2019 and 2020. Otorhinolaryngology and urology were among the most impacted specialties. By 2023, surgical volumes had not recovered, and the deficit remained, except for cholecystectomies, which increased. Effective management is crucial to address the surgical backlog, improve planning, and reduce impacts during future crises.

## Highlights

■ More than 2 million procedures were analyzed in the public health system from 2019 to 2023.
■ A total of 13 types of procedures were performed across 7 different specialties.
■ A 35% reduction in the total number of surgeries was observed.
■ There was no recovery of surgeries postponed during the pandemic.

## Graphical abstract

**Figure:**
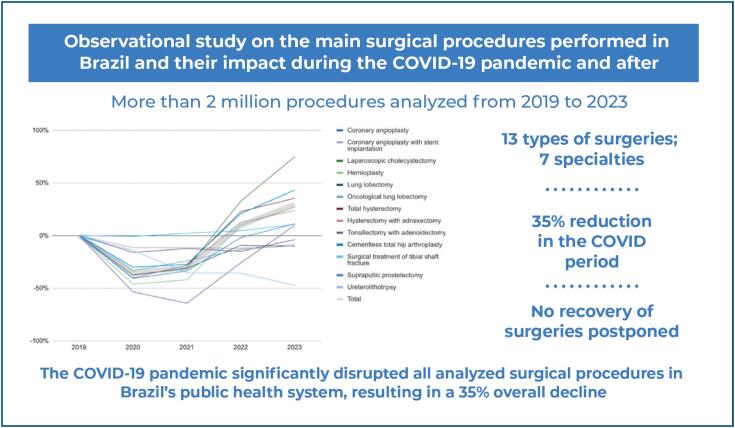

## INTRODUCTION

The Brazilian Unified Health System (SUS - *Sistema Único de Saúde*) is the country's public healthcare system, established under the 1988 Federal Constitution. Article 196 states that "health is everyone's right and the State's duty…," affirming SUS's mission to ensure universal and equitable access to healthcare for all citizens. With a population of approximately 215 million, about 75% rely solely on SUS, totaling over 160 million individuals.^(1)^ In 2024, Brazil's Annual Budget Bill allocated BRL 231 billion to health, translating to a per capita expenditure of about BRL 1,444.75, or approximately USD 267 (based on USD 1 = BRL 5.4). For individuals fully dependent on SUS, the actual per capita annual cost is lower, at roughly BRL 1,074.42 or USD 198.56.^(1)^

Beyond financial constraints, the SUS also faces structural challenges, which were further exposed during the COVID-19 pandemic. The SARS-CoV-2 outbreak, first identified in Wuhan, China, in December 2019, was declared a Public Health Emergency of International Concern by the World Health Organization (WHO) on January 30, 2020, and a global pandemic on March 11, 2020.^(2)^ During periods of peak infection, the sharp rise in hospital admissions caused the temporary collapse of many healthcare systems, including SUS. This overwhelmed capacity limited access to non-COVID-related care and led to widespread postponement of elective surgeries.^(1,3)^

Globally, more than 28.4 million surgical procedures were estimated to have been canceled during the first 12 months of the pandemic.^(4)^ In Brazil, approximately 1.2 million elective surgeries and 210,000 transplants were delayed in 2020 due to hospital overload.^(5)^

Although substantial efforts were made to optimize surgical care during the health crisis, the pandemic's precise impact on overall surgical volume and specific specialties remains unclear. International data show substantial declines in procedural volumes;^(5)^ however, variations in healthcare infrastructure and economic contexts underscore the need for country-specific analyses.^(6)^ As one of the world's largest public health systems, the SUS plays a vital role in delivering surgical services to the majority of Brazil's population. Understanding the pandemic's impact in this setting is crucial for informing recovery strategies and strengthening preparedness for future health emergencies.^(7)^

## OBJECTIVE

To analyze changes in surgical volume across different specialties in Brazil before, during, and after the COVID-19 pandemic.

## METHODS

This descriptive observational study was conducted in São Paulo over a 6-month period in 2024.

Data were sourced from the "TabNet" platform, provided by the Department of Informatics of the Unified Health System (DATASUS - *Departamento de Informação e Informática do SUS*), which contains records of surgical procedures performed within the SUS.

Procedures with the highest annual surgical volumes from 2019 to 2023 were selected, as presented in table 1. Additionally, lung lobectomy was included to represent a highly complex procedure in thoracic surgery.

**Table 1:** Selected surgical procedures

Specialty	Procedure	Procedure code
Cardiology	Coronary angioplasty	0406030014
	Coronary angioplasty with stent implantation	0406030030
General Surgery	Laparoscopic cholecystectomy	0407030074
	Hernioplasty	0407040102
Thoracic Surgery	Lung lobectomy	0412050048
	Oncological lung lobectomy	0416110010
Gynecology	Hysterectomy with adnexectomy	0409060119
	Total hysterectomy	0409060135
Otorhinolaryngology	Tonsillectomy with adenoidectomy	0404010032
Orthopedics	Cementless total hip arthroplasty	0408040092
	Surgical treatment of tibial shaft fracture	0408050500
Urology	Suprapubic prostatectomy	0409030023
	Ureterolithotripsy	0409010596

To access the data, the following steps were taken using the DATASUS platform - https://datasus.saude.gov.br/

1. Navigated to "*Acesso à informação*" (Access to Information) and selected "*Informações de Saúde* (TABNET)" [Health Information (TABNET)].
2. Chose "*Assistência à Saúde*" (Health Assistance) followed by "*Produção Hospitalar* (SIH/SUS)" [Hospital Production (SIH/SUS)].
3. Selected "*Dados Consolidados Autorização de Internação Hospitalar* (AIH), *por local de internação, a partir de* 2008" (Consolidated Hospital Admission Authorization Data (RD), by hospitalization location, from 2008).
4. Under "*Abrangência geográfica*" (Geographical scope), selected "*Brasil por Região e Unidade da Federação*" (Brazil by Region and State).
5. Under "*Linha*" (Row), selected "*região/unidade de federação*" (region/state).
6. For "*Coluna*" (Column), chose "*não ativa*" (inactive).
7. Under "*Conteúdo*" (Content), selected "*Autorizações de Internação Hospitalar* (AIH) *aprovadas*" (Approved Hospital Admission Authorizations).
8. Chose the period from January 2019 to December 2023 under "*Períodos disponíveis*" (Available Periods).
9. Selected "*todas as categorias*" (all categories) under "*Região*" (Region), "*Unidade de Federação*" (State), and "*Caráter de Atendimento*" (Care Character).
10. Under "*procedimento*" (procedure), selected the appropriate procedure code listed in table 1 (*e.g.*, lung lobectomy - 0412050048).
11. Selected "*Ordenar pelos valores da coluna*" (Sort by column values) and "*tabela com bordas*" (table with borders), then clicked "*Mostra*" (Show).

The variable analyzed was the number of approved "*Autorizações de Internação Hospitalar* (AIH)" for the selected procedures. Data were organized into tables and graphs to evaluate annual trends and compare them to 2019, which served as the pre-pandemic baseline. The percentage variation was calculated using the formula:


[(Current year's value/Previous year's value)-1]×100.

As this is a descriptive observational study, no interventions were performed, and only publicly available data were used. No individuals were at risk of privacy violations or harm due to the conduct of this research. Ethical approval was not required, as the study met the exemption criteria under Resolution No. 510/2016 of the CEP/CONEP system (Research Ethics Committee / National Research Ethics Commission).

## RESULTS

**Figure 1:**
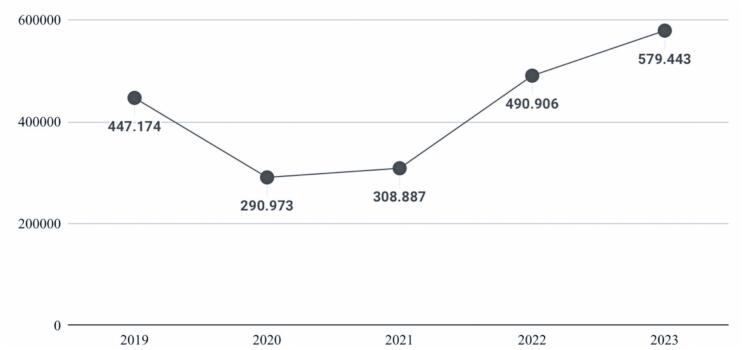
Total approved hospital admission authorization per year

A total of 2,117,383 surgical procedures performed between January 2019 and December 2023 were analyzed, encompassing 13 distinct types of operations (Table 2: total number and annual count by procedure; Figure 1).

**Table 2:** Number of approved *(Autorização de Internação Hospitalar)* per procedure, by year, and total from 2019 to 2023

		2019	2020	2021	2022	2023	Total
Cardiology	Coronary angioplasty	3.503	2.929	3.069	2.978	3.367	15.846
	Coronary angioplasty with stent implantation	51.712	45.758	45.652	45.436	47.361	235.919
General Surgery	Laparoscopic cholecystectomy	93.732	58.544	64.778	124.417	163.968	505.439
	Hernioplasty	122.634	66.135	71.023	134.585	155.931	550.308
Thoracic Surgery	Lung lobectomy	664	414	457	602	597	2.734
	Oncological lung lobectomy	1.231	820	934	1.070	1.113	5.168
Gynecology	Hysterectomy with adnexectomy	31.386	18.777	22.509	35.271	38.907	146.850
	Total hysterectomy	51.399	30.698	36.889	63.123	69.611	251.720
Otorhinolaryngology	Tonsillectomy with adenoidectomy	30.665	14.234	10.950	22.738	33.614	112.201
Orthopedics	Cementless total hip arthroplasty	10.703	7.481	7.779	12.939	15.332	54.234
	Surgical treatment of tibial shaft fracture	33.737	33.435	34.518	35.263	37.303	174.256
Urology	Suprapubic prostatectomy	6.873	4.071	4.556	6.740	7.640	29.880
	Uretero lithotripsy	8.935	7.677	5.773	5.744	4.699	32.828
Total		447.174	290.973	308.887	490.906	579.443	2,117.383

**Figure 2:**
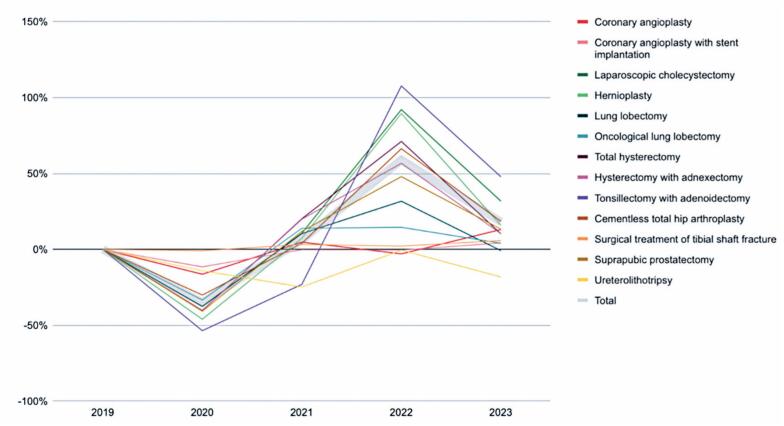
Variations in the number of approved *(Autorização de Internação Hospitalar)* compared to the previous year

A 35% global reduction in surgical volume was observed between 2019 and 2020, with procedure-specific variations ranging from -1% to -54%. By 2022, surgical volume had increased by 10% relative to 2019, and by 2023, the total number of surgeries was 30% higher than the pre-pandemic baseline (Table 3). All specialties experienced a decline in surgical procedures in 2020, with the largest reductions observed in tonsillectomy with adenoidectomy (-54%), hernioplasty (-46%), and hysterectomy with adnexectomy (-40%). In contrast, surgical treatment of tibial shaft fractures showed the smallest decrease (-1%) (Table 3 and Figure 2).

**Table 3:** Variation in the number of approved *(Autorização de Internação Hospitalar)* compared to the previous year

		2020 %	2021 %	2022 %	2023 %
Cardiology	Coronary angioplasty	-16	5	-3	13
	Coronary angioplasty with stent implantation	-12	0	0	4
General Surgery	Laparoscopic cholecystectomy	-38	11	92	32
	Hernioplasty	-46	7	89	16
Thoracic Surgery	Lung lobectomy	-38	10	32	-1
	Oncological lung lobectomy	-33	14	15	4
Gynecology	Hysterectomy with adnexectomy	-40	20	57	10
	Total hysterectomy	-40	20	71	10
Otorhinolaryngology	Tonsillectomy with adenoidectomy	-54	-23	108	48
Orthopedics	Cementless total hip arthroplasty	-30	4	66	18
	Surgical treatment of tibial shaft fracture	-1	3	2	6
Urology	Suprapubic prostatectomy	-41	12	48	13
	Uretero lithotripsy	-14	-25	-1	-18
Total		-35	6	59	18

**Figure 3:**
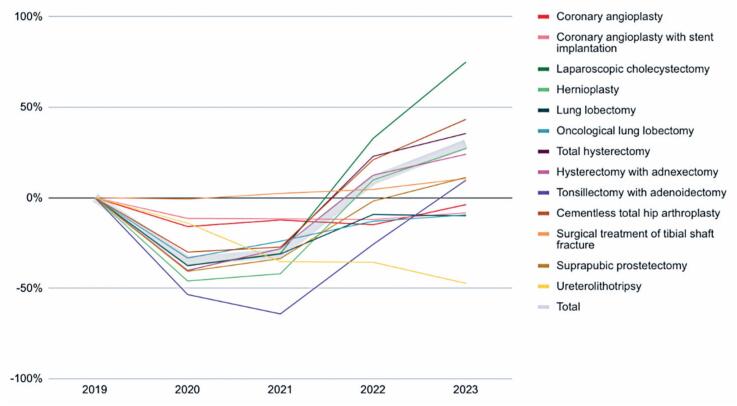
Variations in the number of approved *(Autorização de Internação Hospitalar)* compared to the year 2019

Despite a progressive recovery beginning in 2021, some specialties had not returned to pre-pandemic levels by 2023. Among the procedures that remained below 2019 levels were coronary angioplasty with stent implantation (-8%), pulmonary lobectomy (-10%), and ureterolithotripsy (-47%). In contrast, laparoscopic cholecystectomy (+75%) and total hip arthroplasty (+43%) significantly exceeded pre-pandemic volumes (Table 4 and Figure 3).

**Table 4:** Variation in the number of approved *(Autorização de Internação Hospitalar)* compared to the year 2019

		2020 %	2021 %	2022 %	2023 %
Cardiology	Coronary angioplasty	-16	-12	-15	-4
	Coronary angioplasty with stent implantation	-12	-12	-12	-8
General Surgery	Laparoscopic cholecystectomy	-38	-31	33	75
	Hernioplasty	-46	-42	10	27
Thoracic Surgery	Lung lobectomy	-38	-31	-9	-10
	Oncological lung lobectomy	-33	-24	-13	-10
Gynecology	Hysterectomy with adnexectomy	-40	-28	12	24
	Total hysterectomy	-40	-28	23	35
Otorhinolaryngology	Tonsillectomy with adenoidectomy	-54	-64	-26	10
Orthopedics	Cementless total hip arthroplasty	-30	-27	21	43
	Surgical treatment of tibial shaft fracture	-1	2	5	11
Urology	Suprapubic prostatectomy	-41	-34	-2	11
	Uretero lithotripsy	-14	-35	-36	-47
Total		-35	-31	10	30

The overall recovery in surgical volume did not fully offset the cumulative *deficit* from 2020 to 2021. It is estimated that between 2020 and 2023, 62,864 hernioplasties, 41,124 tonsillectomies with adenoidectomy, and 11,848 ureterolithotripsies were not performed.

### Analysis by specialty

Each specialty demonstrated a distinct pattern during the pandemic. The following section summarizes key observations in each area.

### Cardiology

The analysis included coronary angioplasty and coronary angioplasty with stent implantation.

Coronary angioplasty showed a 16% reduction in 2020, followed by a mild recovery in subsequent years. However, procedure volume in 2023 remained 4% below 2019 levels. The lowest number of procedures occurred in 2020, with 2,929 cases, while the peak was observed in 2019, with 3,503 cases.

Angioplasty with stent followed a similar trend, showing a 12% decline in 2020 and failing to fully recover by 2023, remaining 8% below 2019 levels. The lowest number of procedures was recorded in 2022, with 45,436 cases, while the peak occurred in 2019, with 51,712 cases.

### General surgery

The analysis included laparoscopic cholecystectomy and hernioplasty.

Laparoscopic cholecystectomy saw a 38% drop in 2020 but demonstrated strong recovery, exceeding pre-pandemic levels by 75% in 2023. A total of 505,439 AIH were approved between 2019 and 2023, with the lowest number in 2020 and the highest in 2023.

Hernioplasty declined by 46% in 2020, followed by a gradual increase. However, by 2023, volumes remained 27% below 2019 levels. Across the same period, 550,308 AIHs were approved, with the lowest absolute number in 2020 and the highest in 2023.

### Thoracic surgery

The analysis included pulmonary lobectomy and oncologic pulmonary lobectomy.

Both procedures showed a 30-40% decrease in 2020, followed by partial recovery in subsequent years. By 2023, volumes remained 10% below 2019 levels. For lobectomy, the lowest number of procedures was recorded in 2020, with 414 cases, while the highest was in 2019, with 664 cases. Regarding oncologic lobectomy, there was a 33% reduction compared to 2019, followed by a 9% increase in 2021 (compared to 2020) and an 11% increase in 2022 (compared to 2021). In 2023, volumes were still 10% lower than in 2019, equivalent to 67 fewer procedures, similar to the trend observed in elective lobectomy.

### Gynecology

The analysis included total hysterectomy and hysterectomy with adnexectomy.

Both procedures experienced a 40% reduction in 2020, but showed among the strongest recoveries across specialties, reaching +35% (total hysterectomy) and +24% (hysterectomy with adnexectomy) by 2023. In numerical terms, there were 51,399 authorizations for total hysterectomy in 2019, 30,698 in 2020, and 69,611 in 2023. For hysterectomy with adnexectomy, 31,386 procedures were approved in 2019, 18,777 in 2020, and 38,907 in 2023.

### Otorhinolaryngology

The analysis included tonsillectomy with adenoidectomy.

This was the most affected procedure, with a 54% decline in 2020 and a 64% decline in 2021. The lowest number of surgeries was recorded in 2021, with 10,950 procedures, while the highest occurred in 2023, with 33,614. Recovery was observed in 2022 (+108%) and 2023 (+48%), but by the end of the period, surgical volume was only 10% above 2019 levels, failing to compensate for the cumulative *deficit*.

### Orthopedics

The analysis included uncemented total hip arthroplasty and surgical treatment of tibial shaft fractures.

Total hip arthroplasty declined by 30% in 2020 but recovered significantly, increasing by 43% in 2023 and surpassing pre-pandemic levels. The year with the lowest number of uncemented hip arthroplasties was 2020, with 7,481 procedures, while the highest was in 2023, with 15,332.

Tibial shaft fracture surgery declined by only 1% in 2020, remained stable in the following years, and increased by 11% in 2023. The lowest number of surgeries was recorded in 2020, with 33,435 procedures, while the peak occurred in 2023, with 37,303 procedures.

### Urology

The analysis included ureterolithotripsy and suprapubic prostatectomy.

Ureterolithotripsy was one of the least recovered procedures, with a 47% decline by 2023 compared to 2019. The lowest number of procedures was recorded in 2023, with 4,699 cases, while the highest was in 2019, with 8,935.

Suprapubic prostatectomy declined by 41% in 2020 but showed significant recovery, with an 11% increase in 2023 relative to 2019. The lowest number of procedures was recorded in 2020, with 4,071 cases, while the peak was in 2023, with 7,640.

### Surgical volume recovery

After the return to routine healthcare services, one of the primary challenges was to resume procedures at pre-pandemic volumes. Although some procedures had not yet reached 2019 levels by 2023, most surpassed those values, likely due to accumulated demand and expanded surgical waitlists.

Despite the increase in many procedures beginning in 2021, it remains important to assess whether this growth offsets the decline observed in previous years. In other words, it must be determined whether the surgeries postponed during the pandemic were subsequently performed without compromising the expected procedural volume for the following years.

By 2023, most procedures had still not recovered from the reductions that occurred during the pandemic. The only procedures that recovered or exceeded pre-pandemic levels were laparoscopic cholecystectomy, uncemented total hip arthroplasty, and surgical treatment of tibial shaft fractures. Although most other procedures showed higher volumes in 2023 than in 2019, their cumulative totals remained negatively impacted by the pandemic, with tonsillectomy with adenoidectomy, hernioplasty, and ureterolithotripsy being the most affected.

The total number of surgeries increased by 6% between 2020 and 2021, by 59% between 2021 and 2022, and by 18% between 2022 and 2023. However, a complete recovery from the pandemic's impact has not yet been achieved. The most affected procedures between 2020 and 2023 were hernioplasty, tonsillectomy with adenoidectomy, and ureterolithotripsy, with respective *deficits* of 62,864, 41,124, and 11,848 surgeries. Nonetheless, some procedures showed a positive balance, such as laparoscopic cholecystectomy, which registered 36,780 surgeries above the expected number during the same period.

## DISCUSSION

This study analyzed 2,117,383 surgical procedures performed in Brazil between 2019 and 2023, identifying a 35% decline in total surgical volume in 2020. This finding is consistent with both national and international studies;^(6-10)^ Aguilar et al. reported a 34.1% average reduction in surgical procedures in Brazil through 2021, using a similar methodology.^(11)^ Globally, the impact varied across healthcare systems. In the United States, the reduction was 10.2% between 2019 and 2020, with a partial recovery by the final quarter of 2020.^(5)^ In the United Kingdom, the decline was more pronounced at 33.6%, leading to the cancellation of over 1.5 million surgeries.^(12)^ Although this study focused on only 13 procedures—estimating approximately 300,000 surgeries not performed by 2021—the broader analysis by Aguilar et al. offers a national overview using DATASUS data, indicating that over 1.5 million surgeries were postponed during the same period.^(11)^ Other countries also reported significant variations, though with different magnitudes. In the United States, 13,108,567 procedures were analyzed from 2019 to 2021, with an annual reduction of 10.2% from 2019 to 2020. During the first three months of the pandemic (March to May 2020), a 48% drop in global surgical volume was observed. Later, in the final quarter of 2020, the reduction narrowed to just 3% compared to the same period the previous year, despite the persistence of COVID-19. Overall, 678,348 fewer procedures were performed in the United States in 2020 compared to 2019.^(5)^

In England and Wales, there was a 33.6% reduction in surgical procedures—3,102,674 procedures were performed in 2020 compared to a historical average of 4,671,338. This resulted in the cancellation of over 1.5 million surgeries.^(12)^

Disparities between countries may be attributed to differences in per capita healthcare spending. While the SUS allocates approximately USD 200 per capita annually, healthcare expenditures are 26 times higher in the United Kingdom (USD 5,387) and 62 times higher in the United States (USD 12,555).^(13-15)^ Additionally, the presence of a private healthcare sector that serves approximately 50 million Brazilians may have helped reduce some pressure on the public system, thereby complicating direct comparisons.^(16)^

### Emergency *versus* elective procedures

International studies have reported significant reductions in emergency procedures, largely attributed to decreased hospital visits and increased use of conservative management strategies during the pandemic.^(12,17-19)^ This study observed substantial declines in angioplasty, cholecystectomy, and hernioplasty, which align with this global trend. In contrast, surgical treatment of tibial shaft fractures remained stable (+6% in 2023), possibly due to increased demand for motorcycle deliveries, as motorcyclists are a high-risk group for such injuries.^(20)^

Among elective procedures, tonsillectomy with adenoidectomy was the most affected, with a 64% reduction in 2021 and only partial recovery by 2023 (+10% compared to 2019). Similar patterns were observed in Germany (-76%) and the United States (-70%), suggesting an additional impact of social isolation measures on pediatric respiratory infections.^(21)^

Elective procedures were the most impacted during the pandemic due to their non-emergency nature and the strategic decisions made by healthcare services. Several studies have reported a sharp reduction in the volume of elective surgeries,^(6,11)^ many of which were either canceled or postponed, potentially compromising patient health by increasing the likelihood of more complex future interventions and expanding surgical waiting lists. Similarly, all selected elective procedures in this study experienced volume declines (Figure 3).

### Impact on surgical capacity and backlog

Although recovery began in 2021, increased surgical volumes did not compensate for the backlog accumulated in 2020 and 2021. It is estimated that 62,864 hernioplasties, 41,124 tonsillectomies with adenoidectomy, and 11,848 ureterolithotripsies were not performed during this period. Studies suggest that with a 20% increase in surgical capacity, it would take 11 months to eliminate a 3-month backlog of procedures.^(22)^ However, the pace of recovery within the SUS was slower, likely due to resource limitations and elevated post-pandemic demand.

A similar decrease in the number of hernioplasties was observed in other countries. In Canada, a study comparing surgical volumes in 2020 *versus* 2019 reported 60,038 elective and 23,173 urgent inguinal hernioplasties.^(23)^ The data revealed a 21% reduction in elective procedures, resulting in a *deficit* of 3,517 surgeries, and a 17% decrease in urgent procedures, with a *deficit* of 1,049 surgeries.^(23)^ In the present study, although the data did not distinguish between elective and urgent hernioplasties, a greater overall reduction of 46% was observed from 2019 to 2020. Additionally, the Canadian study, which presented data by month, showed clear fluctuations in surgical admissions corresponding to the first and second COVID-19 waves.^(23)^ In contrast, as this study used annual data, such fluctuations were less apparent.

Urology was another area significantly affected worldwide. Although complications of ureterolithiasis are considered emergencies, a marked decrease in the volume of ureterolithotripsy was observed during and after the pandemic. A similar pattern was reported in other countries. For example, a study conducted in Italy detected a 55% reduction in urological emergencies between 2019 and 2020,^(24)^ with consistent findings reported in regions such as Poland, Dallas, and Saudi Arabia.^(25-27)^ Steinberg et al. proposed that there may have been overtreatment of renal colic before the pandemic,^(26)^ while Militaru et al. suggested that delays in patients seeking medical care during the pandemic, along with the increased use of alternatives such as ureteral stents, contributed to the observed decline in urological emergency procedures.^(28)^

Regarding cholecystectomy, a decline in the number of procedures between 2019 and 2020 was also reported in other countries. In the aforementioned Canadian study, elective cholecystectomies decreased by 61%, and emergency cholecystectomies declined by 8%.^(23)^ A similar reduction was observed in Japan during the same period,^(29)^ aligning with the 38% decline reported in this study. However, this study also analyzed the post-pandemic recovery in procedure volumes. By 2021, an estimated *deficit* of 60,000 cholecystectomies was recorded, consistent with earlier reductions. This *deficit* was nearly halved by 2022 and fully reversed by 2023, when more than 30,000 procedures were performed beyond the expected number. Hamid et al. employed a strategy that included multi-hospital coordination, mobile operating rooms, and a 7-day surgical schedule. Implementing similar measures, such as extended surgical hours, mobile surgical centers, and coordinated hospital networks, may be essential for optimizing recovery. International experiences further suggest that approaches like scheduled weekend surgeries can reduce wait times from 428 to 49 days.^(30)^

### Study limitations

This study has some limitations. First, it is restricted to data from the public health sector and does not include information from the private system. Second, the database did not distinguish between emergency and elective procedures, limiting more granular analysis. Third, the study did not assess the clinical impact of delayed surgeries. Future research should incorporate private sector data and evaluate long-term clinical outcomes to better understand how reduced surgical volume affects population health. Additionally, further studies should investigate the clinical consequences of reduced surgical activity and identify effective strategies to restore surgical capacity in resource-limited healthcare systems.

## CONCLUSION

The COVID-19 pandemic significantly impacted surgical volume within the Brazilian Unified Health System, resulting in a 35% overall reduction in 2020. Although a progressive recovery began in 2021, the increased number of procedures was insufficient to fully offset the backlog accumulated in earlier years.

Furthermore, for most procedures evaluated, the accumulated deficit was not recovered, which implies prolonged wait times and potential consequences for population health in Brazil.
